# TwinPort: 5G drone-assisted data collection with digital twin for smart seaports

**DOI:** 10.1038/s41598-023-39366-1

**Published:** 2023-07-29

**Authors:** Yagmur Yigit, Long D. Nguyen, Mehmet Ozdem, Omer Kemal Kinaci, Trang Hoang, Berk Canberk, Trung Q. Duong

**Affiliations:** 1 Department of Computer Engineering, Faculty of Computer and Informatics, Istanbul Technical University, Istanbul, Turkey; 2 Duy Tan University, Da Nang, Vietnam; 3 Innovation and Product and Service Development Directorate, Turk Telekom, Ankara, Turkey; 4 Department of Shipbuilding and Ocean Engineering, Istanbul Technical University, Istanbul, Turkey; 5 Ho Chi Minh City University of Technology (HCMUT) - Vietnam National University VNU-HCM, Hanoi, Vietnam; 6 School of Engineering and Built Environment, Edinburgh Napier University, Edinburgh, UK; 7 School of Electronics, Electrical Engineering and Computer Science, Queen’s University Belfast, Belfast, Northern Ireland

**Keywords:** Electrical and electronic engineering, Environmental impact

## Abstract

Numerous ports worldwide are adopting automation to boost productivity and modernize their operations. At this point, smart ports become a more important paradigm for handling increasing cargo volumes and increasing operational efficiency. In fact, as ports become more congested and cargo volumes increase, the need for accurate navigation through seaports is more pronounced to avoid collisions and the resulting consequences. To this end, digital twin (DT) technology in the fifth-generation (5G) networks and drone-assisted data collection can be combined to provide precise ship maneuvering. In this paper, we propose a DT model using drone-assisted data collection architecture, called TwinPort, to offer a comprehensive port management system for smart seaports. We also present a recommendation engine to ensure accurate ship navigation within a smart port during the docking process. The experimental results reveal that our solution improves the trajectory performance by approaching the desired shortest path. Moreover, our solution supports significantly reducing financial costs and protecting the environment by reducing fuel consumption.

## Introduction

Seaports are the backbone of the global economy and play an essential role in international trade. Many ports worldwide are turning to automation to increase efficiency and improve their operations to keep up with technological advancements^1^. As such, the concept of “smart ports” has recently become more focused. The smart port market is anticipated to expand rapidly at a rate of 24.3%, from an expected 1.9 billion in 2022 to a total value of 5.7 billion USD by 2027^2^. This evolution will be feasible only with the further application of intelligent solutions such as big data analytics, artificial intelligence (AI), Internet-of-Things (IoT), and more to the seaport environment.

One area of seeing the potential of these technologies is ship maneuvering. At active seaports, precise ship maneuvering is critical in navigating through tight or crowded port zones. Delays resulting from inaccurate ship navigation can significantly impact shipping schedules, leading to missed deadlines and financial loss for shipping companies. Failure to apply the appropriate actuator actions could result in ships colliding with one another, leading to potential harm or loss of life. Furthermore, operating a vessel through confined waters such as seaport entries requires significant expertise and high attention. If proper maneuvering techniques are not applied, ships may collide with others, ultimately causing damage or even death. Furthermore, operating a vessel through a seaport requires significant expertise and skill that all ship captains may not have. Therefore, automated solutions are essential to provide precise ship maneuvering. Moreover, precise ship maneuvering can positively impact the environment by reducing carbon dioxide emissions. Automated solutions can significantly reduce the fuel consumed and emissions generated by decreasing the distance ships travel in congested seaports.

Digital twin (DT) and drone-assisted data collection technologies can be combined to provide precise ship maneuvering. DT is a promising technology with broad application fields, such as real-time or right-time remote monitoring and management, predictive maintenance, and more^3^. In this technology, digital replicas of physical entities are created and used for several objectives, such as optimization, fast simulation, and performance testing^4^.

In the fifth-generation (5G) networks and beyond, unmanned aerial vehicles (UAVs), also called drones, have comprehensive coverage and great mobility. Drone-assisted data collection is advantageous for many reasons: drones are highly agile, have high flexibility and low cost, and can collect data near sensors, significantly reducing the energy consumption of IoT. Furthermore, drones can be used as on-demand access points with aerial prominence. They are able to supply suitable line-of-sight routes for IoT entities. Thus, they enhance the quality of service, leading to better wireless communications than classic techniques^5^. Drones are a more economical solution to collecting IoT data in remote areas that do not have terrestrial infrastructure, compared with conventional alternatives such as long-range ground broadcasting stations or communication via satellites, which often come with high costs.

**Figure 1:**
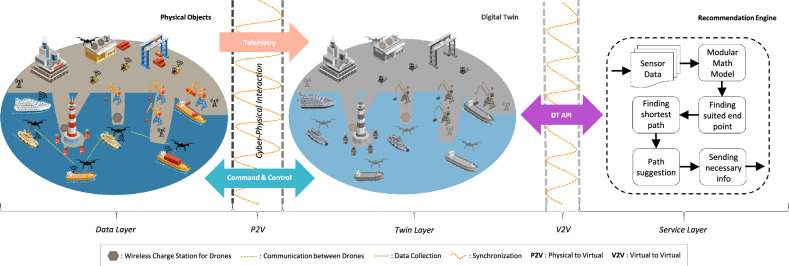
Proposed 5G digital twin architecture for smart seaports.

### Related works

Data collection with UAVs or drones has recently become one of the most popular research topics. Caruso *et al.* conducted an analytical study to determine how near the sensors a drone using a long-range (LoRa) radio needs to fly to collect data for applications requiring precision agriculture^6^. Wei *et al.* thoroughly investigated the methods and technologies of UAV-assisted data collection for IoT^5^. Yuan *et al.* introduced an approach to optimize completion time by simultaneously determining the UAV trajectory and the sensor network assignment scheme to assist data collection from multiple sensor nodes^7^. The research by Liu *et al.* examined the UAV trajectory planning issue in an environmental monitoring system^8^. In the study, a typical data collection scenario is considered, in which a UAV is sent to a specific location to collect time-sensitive data in several monitoring areas and transmit the data to a ground base station.

UAVs are extensively being used in numerous industries, including the maritime sector. Nevertheless, the idea of utilizing IoT systems to enhance the maritime sector’s intelligence is a relatively new concept. Therefore, only a few studies have focused on this topic. For instance, Liang *et al.* studied multi-UAV marine IoT systems that use UAVs as aerial base stations to gather data from buoys and transfer it to an offshore base station^9^. Chapapria *et al.* investigated developing coastal monitoring systems using UAVs that enable data collection, approaches incorporating image processing and computer vision algorithms for analysis, and marine data^10^. In another study, a multi-UAV-enabled maritime communication paradigm was put forth in which UAVs transmit data for marine users^11^. The power allocation, UAV trajectory, and user association are jointly optimized to maximize the lowest average throughput among all users to enhance the communication systems’ performance.

On the contrary, DT is quite a new concept in the maritime sector, and the literature only consists of recent works in this field. Examining the security performance of marine transportation systems when utilizing IoT and DT technologies, Liu *et al.* presented a model for marine DTs based on IoT relay cooperation. The simulation verified the proposed model’s security and transmission performance.^12^. Also, on the security topic, in our previous work, we recommended a DT-assisted honeypot called TwinPot with an advanced attack detection system to handle various attack types for smart seaports^13^. Other than the aforementioned, DT is being used by marine engineers to enhance and optimize ship operations. These include estimation of speed loss due to marine fouling^14^, tracking ship life cycle^15^, and aid ship maneuvering to acquire full navigation automation^16^.

### Coverage of article and contributions

As aforementioned, plenty of works have focused on data collection, monitoring, and inside port operations using UAVs in different aspects. Meanwhile, only a few works focus on using DT in the maritime domain. Moreover, none of those mentioned works addressed the topic of precise ship motion for smart seaports. An automated management system minimizes ship delays and downtime in smart seaports using IoT, DT, and drone-assisted data collection. Furthermore, when docking into a smart seaport, precise ship motion can be provided.

Our main contributions to this paper are as follows:

- We propose a DT model using drone-assisted data collection architecture, called TwinPort, to provide a comprehensive management system deploying IoT, DT, and drone-assisted data collection technologies for smart seaports.
- We introduce a basic data collection protocol using IEEE 802.11p to communicate between ships and drones.
- We offer a communication methodology between drones inspired by a self-organizing network (SON).
- We suggest a recommendation engine for precise ship motion when ships dock in a smart seaport.

**Figure 2:**
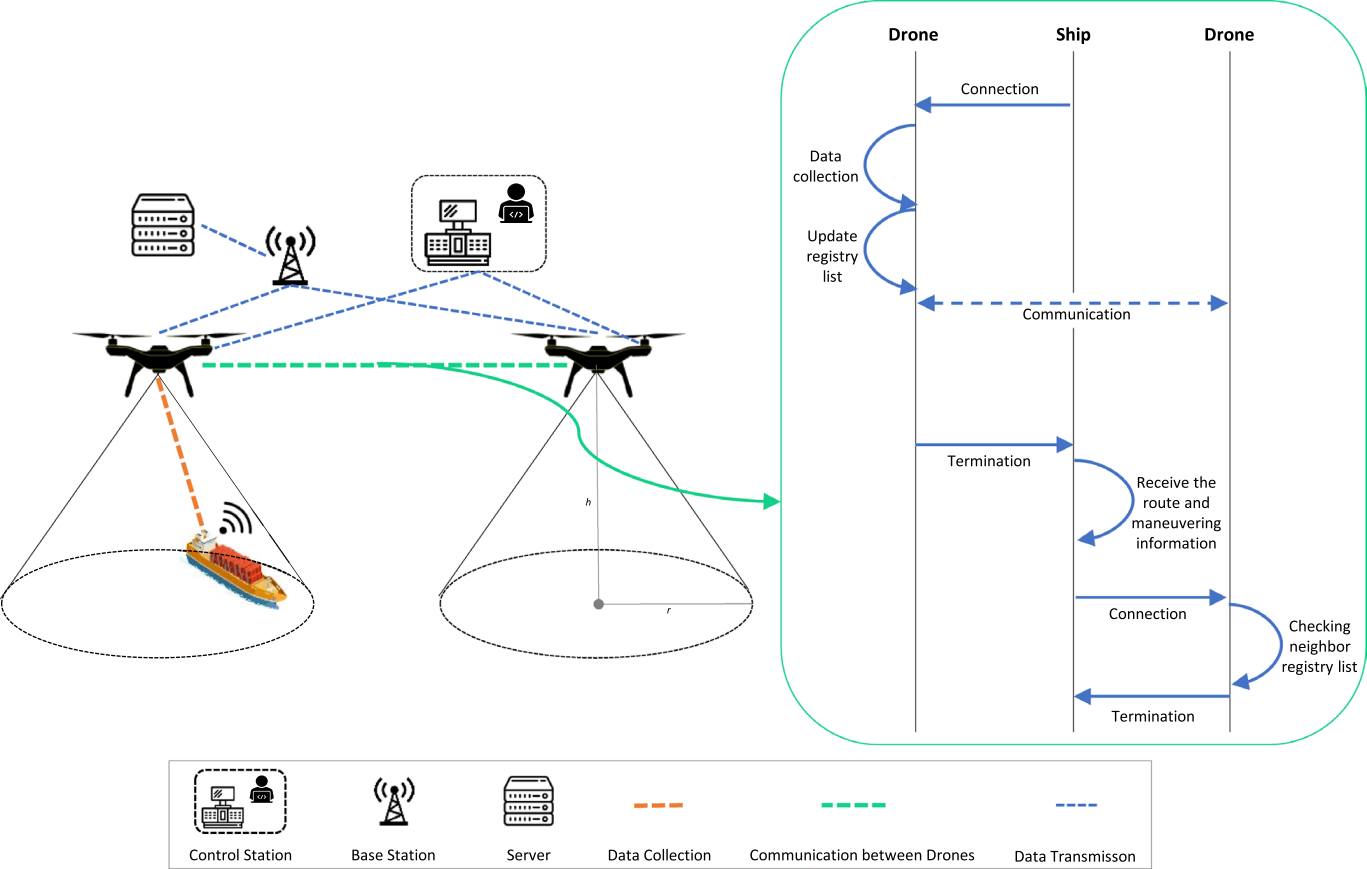
Drone-assisted data collection network and the communication diagram between drones.

### Preliminary study

Before beginning our implementation, we scrutinized the effect of DT technology on a ship’s path. We investigated a 320 m-long container ship that needed to follow a predefined course in two cases. In the first case, it uses only sensors to define the trajectory; and in the second, the combination of DT and sensors is used. We created a simple system with a start point, an endpoint, and two waypoints. The shortest distance that needs to be covered by the ship is 8850 m, and the ship must pass all the stations.

In the first case, the 9394 m distance was covered in 30.3 minutes. On the contrary, 8878 m was covered in 29.3 minutes when sensors and DT technology were used together. It indicates that the ship completed the track 1 minute earlier, and the covered distance was shorter by about 500 meters. Furthermore, the container ship has a main engine of roughly 40,000 kW. During that additional one minute, it would consume 2.4BJ of energy, corresponding to 65 liters of diesel oil at the energy volume of 36.9 MJ per liter^17^. Thus, the combination of sensors and DT can save 3200 liters of diesel oil per day per ship. Considering the financial benefits of this, it is 270 USD per hour, 6.48 thousand USD per day, and 2.3 million USD per year that can be saved^18^.

As can be seen from our preliminary experiment, our proposed solution can decline financial expenditure significantly and consumption of fuel that protects the environment by reducing carbon emissions.

The rest of the paper is organized as follows: First, we propose and explain the system model. Next, we describe the performance evaluation of our solution. And then, we provide a discussion of the proposed study and the paper’s conclusion.

## Proposed system model

We propose a three-layered approach, which includes physical-to-virtual (P2V) and virtual-to-virtual (V2V) communications, to create a comprehensive DT architecture for smart seaports. Our proposed architecture aligns with the Gemini Principle, especially considering the global net-zero directions and actions^19^. We incorporate the essence of the Gemini principle, focusing on quality, evolution, and insight values across all layers of the digital twin in developing our architecture. Figure 1 depicts the proposed 5G drone-assisted data collection system using DT for smart seaports.

The first layer, *Data Layer*, is comprised of seaport physical entities, such as IoT devices, which generate real-time data. This layer is the foundation of the digital twin architecture as it enables the collection and transmission of data to the subsequent layers.

The second layer is *Twin Layer*, which gives digital replicas of the physical entities. The Twin Layer is responsible for modeling and simulating the physical seaport. It enables stakeholders to monitor and analyze seaport operations.

*Service Layer*, the third layer, includes smart seaport applications, such as a recommendation engine and maneuver forecasting. This layer uses data that comes from the twins created in the Twin Layer to provide insights and predictions on seaport operations. Overall, the proposed three-layered architecture for smart seaports’ digital twins provides a comprehensive solution for monitoring, analyzing, and optimizing seaport performance. Our solution provides practical insights into seaport processes and enhances seaport efficiency and sustainability.

### Drone-assisted data collection

This data collection method provides several benefits and abilities, including improved safety, enhanced efficiency, cost savings, and real-time data collection in smart seaports. It can revolutionize ship maneuvering processes and improve precision and efficiency during ship maneuvering. It is especially critical for container ships’ entry and departure processes at a seaport. Our proposed solution uses drone-assisted data collection to enhance precision and efficiency during ship maneuvering.

The components of the primary drone-assisted data collection network include sensors, a drone, a control station, a base station, and a server, which are described below:

- The sensors discern the environment according to their unique properties and send data to the drone.
- The drone collects data while flying over the ships, then transmits it to the server.
- The control station automatically designs the drone’s flight path to optimize data collection.
- The base station receives sensing data from the drone.
- The server stores and sends the data obtained from the sensors to the connectivity layer.

The data is stored on both the server physically and the service layer digitally.

**Figure 3:**
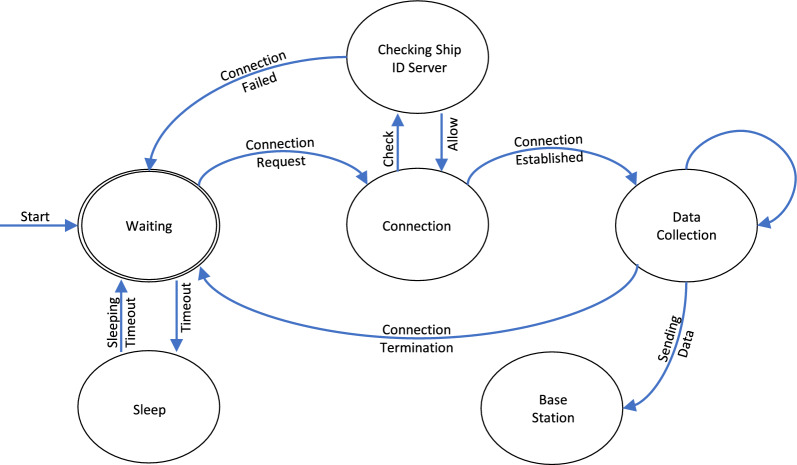
The state transition diagram for the communication between ships and drones.

Figure 2 gives the basic network architecture of drone-assisted data collection. *r* represents the radius of the data collection area, and *h* depicts the drone’s altitude. *r* depends on *h* and this relation changes according to sensor types. Some sensors need close data collection, while others do not require the same distance. Therefore, *h* needs to be specified to optimize the distances to sensors.

The altitude limit for drones near seaports varies depending on the location and regulations in the particular area. The legal height limit is 400 feet for near seaports in the UK, according to the Civil Aviation Authority (CAA); hence the maximum altitude of drones in our model is 400 feet. Moreover, the drones are positioned such that the intersection of each other’s data collection areas is avoided.

In our proposed system, drones collect data in hovering mode. Moreover, we used the Hilbert curve, which is a widely used graph theory-based path planning algorithm to define drone flight since we used a graph database for twins^20^. Since we focus on precise ship maneuvering in this work, we take into account only gathering the necessary details of the ship during the data collection process, such as length, breadth, draft, and more. To this end, we defined a basic data collection protocol using IEEE 802.11p for the communication between ships and drones. The IEEE 802.11p standard is a wireless communication protocol designed specifically for vehicular environments^21^. Figure 3 depicts the state transition of the proposed protocol for ship and drone communication.

- *Waiting state*: The drone is waiting for a ship to initiate communication. The drone remains idle until it receives a signal from a ship. The drones remain in standby mode and conserve energy until they receive a connection request from a ship during this state.
- *Connection state*: When the drone receives a signal from a ship, it establishes a connection with the ship. A reliable communication link is established between the drone and the ship, thanks to this state.
- *Checking ship ID server state*: The drone authenticates the identity of the ship accessing the ID Server. This server has data about registered ships as well as a blacklist. The drone verifies that the requested communication is authorized and not blacklisted.
- *Data collection state*: When the identity of the ship is confirmed, the drone enters the data collection state. In this stage, the drone collects the necessary information on the ship at this stage, including its length, breadth, draft, and other relevant information. This process is essential for the precise maneuvering of the ship.
- *Base station state*: When the data collection process is over, the drone sends the data to a base station. The base station operates as a central node for the analysis and storage of data.
- *Sleep state*: Once the data has been transferred, the drone goes to sleep. Until it gets another signal from a ship to start the communication process once more, it is dormant.

These states explain how communication between ships and drones for data collecting progresses sequentially. The proposed approach enables precise ship navigation by ensuring the necessary information is efficiently and accurately gathered.

#### Communication between drones

An Automatic Neighbor Relation (ANR) mechanism in our system, inspired by the concepts of Self-Organizing Network (SON) architecture, facilitates communication between drones. By enabling drones to form and manage neighbor relationships autonomously, SON offers a decentralized and intelligent method to network management. This results in automated discovery and effective communication between network elements. ANR is essential for providing seamless communication between nearby drones, facilitating information sharing, and improving overall network performance.

The essential component of the system is each drone’s registration, which contains a list of the details of the ships from which it gathers data. Before beginning any data-gathering operations, the drone can proactively verify its neighbor drone’s register list thanks to the ANR system. By taking this crucial step, the drone may efficiently manage the generation of new routes for ships that have already been addressed and prevent redundant data collecting from the same ship. Several critical steps make up the communication process between drones and the function of ANR in the proposed system:

- *Neighbor drone discovery*: Each drone in the system is aware of the drones around it. This information is essential to building an interconnected drone network and establishing effective communication.
- *Intersect-free data collection zones*: To avoid interference and conflicts, the data gathering areas for nearby drones are not intended to cross paths. The smooth operation of drones without interfering with another one’s data collection operations is ensured through elaborate planning.
- *Communication link establishment*: The communication links between the nearby drones are created after they have been recognized as such. These connections provide the framework for the exchange of data and registry details, facilitating seamless communication between drones.
- *Data collection process*: The process of data collecting begins when a ship enters the drone’s data collection zone. Based on its predetermined settings and objectives, the drone begins collecting data from the ship.
- *Updating registry lists*: The drone updates its registry list with the relevant ship’s information after the data collection operation is over. The drone is guaranteed to remain aware of the ships from which it has already gathered information thanks to this.
- *Registry list exchange*: Through the established communication links, the neighbor drone or drones receive the updated registry list of each drone. By exchanging information, nearby drones can keep track of another one’s data collection efforts.
- *Neighbor registry check*: When a ship enters the data collection zone of a neighbor drone, the drone checks its neighbor’s registry lists. If the ship’s record is found in any neighbor’s registry list, it indicates that the ship has already been addressed. Consequently, the drone terminates the connection of data collection for that ship, preventing duplication of efforts.
- *Periodic registry list management*: The registry lists are periodically deleted or refreshed to prevent overloading and maintain the most up-to-date information. This maintenance step helps drones to focus on relevant and recent data sources while managing memory efficiently.

The flow of this process is illustrated on the right side of Fig. 2. By employing the SON architecture and ANR mechanisms, the proposed system enables effective communication and seamless collaboration between drones, optimizing data collection efforts and avoiding duplication of work in dynamic and changing environments. This autonomous and intelligent approach helps improve the overall efficiency and reliability of the data collection process in the context of ship monitoring and maneuvering.

### Proposed recommendation engine for precise ship maneuvering

Conventional ship maneuvering methods often rely on manual decision-making by master mariners or basic navigation systems, which may not fully leverage the available data and advanced mathematical models to optimize the ship’s path. Therefore, we proposed a recommendation engine that introduces a novel method for ship maneuvering by leveraging data from a twin layer, constructing a modular mathematical ship model, and employing path-following algorithms. The primary rationale behind our proposed recommendation engine is to provide automated and data-driven recommendations for the optimal path and maneuvering specifications of ships as they enter or leave ports. It builds a modular mathematical ship model using the twin-layer data, allowing for accurate computations and analysis. The system finds the best location for the lineup procedure and calculates the quickest and most appropriate path by taking into account variables like open slots in the ports and the ship’s features. This data-driven approach helps avoid unnecessary route duplications and ensures efficient use of available port resources.

The core workflow of the proposed recommendation engine is as follows:

- The required data is taken from the twin layer.
- The modular mathematical model of the ship is built. (We used the same formulation for the modular mathematical model in our previous work^22^. Still, a brief introduction is given below for clarity.)
- Checking empty slots in the seaport according to the ship’s properties.
- Defining the best appropriate area for the lineup process of the ship.
- The shortest path named the desired path is determined.
- Then, the actual path is calculated.
- The specifications are created according to the actual path for precise maneuverings such as heel angle, drift angle, speed, roll velocity, and more.
- After that, these specifications are sent to the master mariner.
- The best area of the ship is held until the decision.
- If the master mariner accepts the recommended route, the reserved port area is signed as busy in the system.
- On the other hand, if the master mariner does not accept the recommended route, the reserved port area is signed as empty in the system.
- We define a specific time slot for this decision process. If the system does not get a response from the related ship, the reserved port area is signed as empty.

The above process describes taking a ship into port. The same procedure is followed for the outbound process. The ship maneuvering model is based on the MMG model that considers the hull, propeller, and rudder separately. For a ship assumed to have three degrees of freedom, the model can be mathematically stated by equation (1).

1 $$\begin{aligned} \begin{aligned} m [{\dot{u}} - rv - x_{G}r^{2}]&= X_{H} + X_{R} + X_{P} \\ m [{\dot{v}} + ur + x_{G}{\dot{r}}]&= Y_{H} + Y_{R} \\ I_{z}{\dot{r}} + mx_{G}({\dot{v}} +ur)&= N_{H} + N_{R} \end{aligned} \end{aligned}$$

In equation (1), *X* and *Y* are the surge and sway forces, and *N* is the yaw moment. Sub-indices *H*, *R*, and *P* denote the hull, rudder, and propeller, respectively. *u* and *v* are surge and sway velocities, *r* is the yaw velocity, and the dots denote the time derivative (in this case, they refer to acceleration). *m* is the displacement tonnage of the ship, ($x_{G}$) center of gravity, and ($I_{z}$) moment of inertia around the yaw axis. The hydrodynamic interaction between the hull and the appendages is handled by the modular structure of the model. The waypoint navigation is realized by the path-following algorithm that implements PID control.

## Performance evaluation

we designed a simulation setup that closely mimics the real-world scenario to evaluate the performance of our proposed digital twin model for precise ship maneuvering. This section explains our simulation setup and then presents the performance result. The architecture built in this study may be used in confined waters such as straits, channels, or rivers where there is a need for precise ship maneuvering. The ship is commanded by a controller in its digital twin, which uses its actuators (the rudder and the propeller) to turn and change the ship’s speed. In this respect, we have investigated the situation of the ship entering the Port of Leith. To conduct our experiment, we defined the data layer location as the Port of Leith, Scotland’s largest enclosed deep-water port. This port can handle ships up to 50,000 deadweight tonnes and manage over 1 million tonnes of cargo^23^. In addition, its complex geographical features, such as narrow channels, were also influential in picking this port.

In what follows, we will describe our simulation environment. To this end, we have defined a particular ship that will dock at the Port of Leith to conduct our experiment. The geometric details of the investigated ship can be seen in Table 1. Geometric details of the ship are crucial as they affect the fuel consumption and finding the most suitable area for the sorting process. Therefore, we ensure that this information is imported into the system by using our proposed protocol in the drone-assisted data collection process.

**Table 1:** The geometric details of the investigated ship.

Name	Unit (m)
Length	180 m
Breadth	32.22 m
Draft	8.19 m
Block coefficient	0.547
Longitudinal center of gravity	-2.52 m
Vertical center of gravity	13.5 m
Metacentric height	2.52 m
Propeller diameter	5.85 m

**Figure 4:**
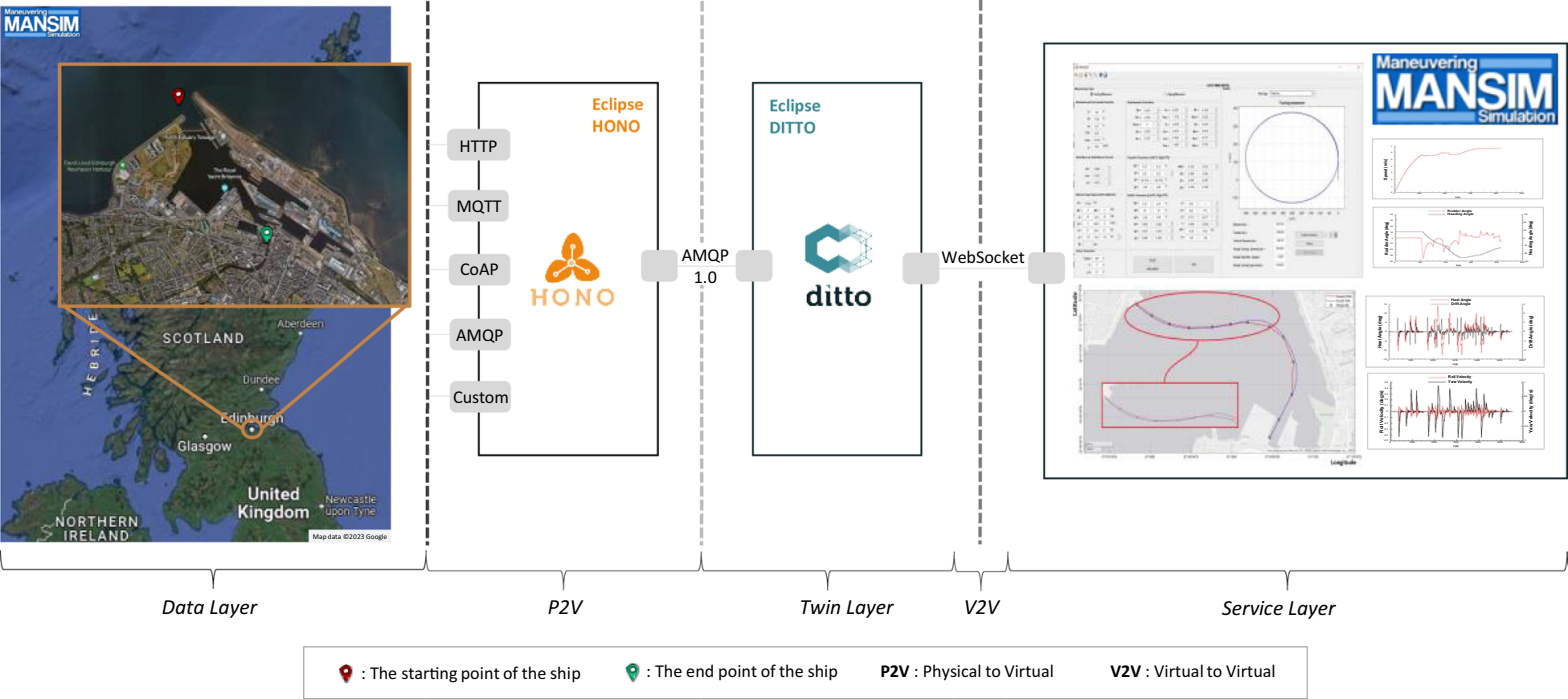
The simulation architecture.

### Simulation environment

We used Eclipse Hono, Eclipse Ditto, and MANSIM tools, all of which are open source, to build a comprehensive simulation. Eclipse Hono submits remote service interfaces connecting many IoT or physical entities to a subsequent layer. It also consistently interacts with them regardless of the assets’ communication protocol. Eclipse Ditto is an open-source framework that delivers a scalable and flexible solution for building digital twins of physical entities^24^. The MANSIM is a code for maritime simulation that produces time-dependent ship motion information for ships^25^. In the data layer, we also employed the extended MANSIM version 2.01 to take geographical data of the port and send data. The MANSIM tool uses Google Maps^26^ to take satellite imagery and geographical data. Figure 4 shows the architecture of our simulation setup, which consists of three layers.

We defined the starting and end points for the ship in the data layer, representing the real-world environment using the MANSIM. We employed Eclipse Hono version 2.2.0 between the first and second layers to send data from the physical assets to the second layer. Eclipse Ditto version 3.0 were used for digital replicas of physical assets in the twin layer. Lastly, we extended the MANSIM version 2.01 by adding the WebSocket feature and the proposed recommendation engine. We utilized this tool in the service layer.

We created *“Drone1”* and *“Ship1”* as things, adding their features and attributes in the Eclipse Ditto. Then, we built the connection between the Eclipse Ditto and the extended MANSIM using WebSocket. Meanwhile, the link was created between Eclipse Hono and Eclipse Ditto using the Advanced Message Queuing Protocol (AMQP) version 1.01.

We used ns-3 network simulator version 3.29 to test the performance of the proposed protocol for ship and drone communication^27^. For the data transmission measurements, we employed version 2 of the ns-3 network performance tool^28^. The data was transmitted at a rate of 50kbps, with a packet size of 128 bytes. The simulation lasted for 120 seconds, and the transmission process commenced during the fifth second of the simulation period.

### Results

Table 2 shows the performance results of the proposed protocol for ship and drone communication. As can be understood from the result, the protocol performance is at a level to provide data collection.

**Table 2:** The performance results of the proposed protocol for the communication between ships and drones.

Number of ships	End to end delay (ms)	Packet delivery ratio
1	22.594	0.993
2	28.365	0.987
3	34.086	0.968
4	38.516	0.927
5	42.423	0.923

We produced ship data manually and sent it to the Eclipse Hono using the Message Queuing Telemetry Transport (MQTT) adapter. Then, the data was transmitted to the second layer using the AMQP protocol. After that, it was transferred to the MANSIM using the WebSocket. The specified slot in the seaport can be seen in the data layer in Fig. 4.

In our simulation, the physical entity, Ship1, was moving in a water environment, and we were tracking its movement and attributes. We can accurately simulate the ship’s motion, including its speed, direction, and acceleration, using the MANSIM. Drone1 monitored the ship’s movement and collected data about the ship’s surroundings. The data collected by Drone1 was sent to Eclipse Hono, which was then forwarded to Eclipse Ditto. Eclipse Ditto then used this data to update the digital twin of Ship1. The recommendation engine produced the desired and actual paths based on the data collected by Drone1 and DT of Ship1. Next, it generated automated maneuvering data according to the actual path and sent it to the crew of Ship1. By utilizing the recommendation engine, the staff of Ship1 can make informed decisions, which can help to prevent accidents and improve the overall efficiency of the ship’s operations.

**Figure 5:**
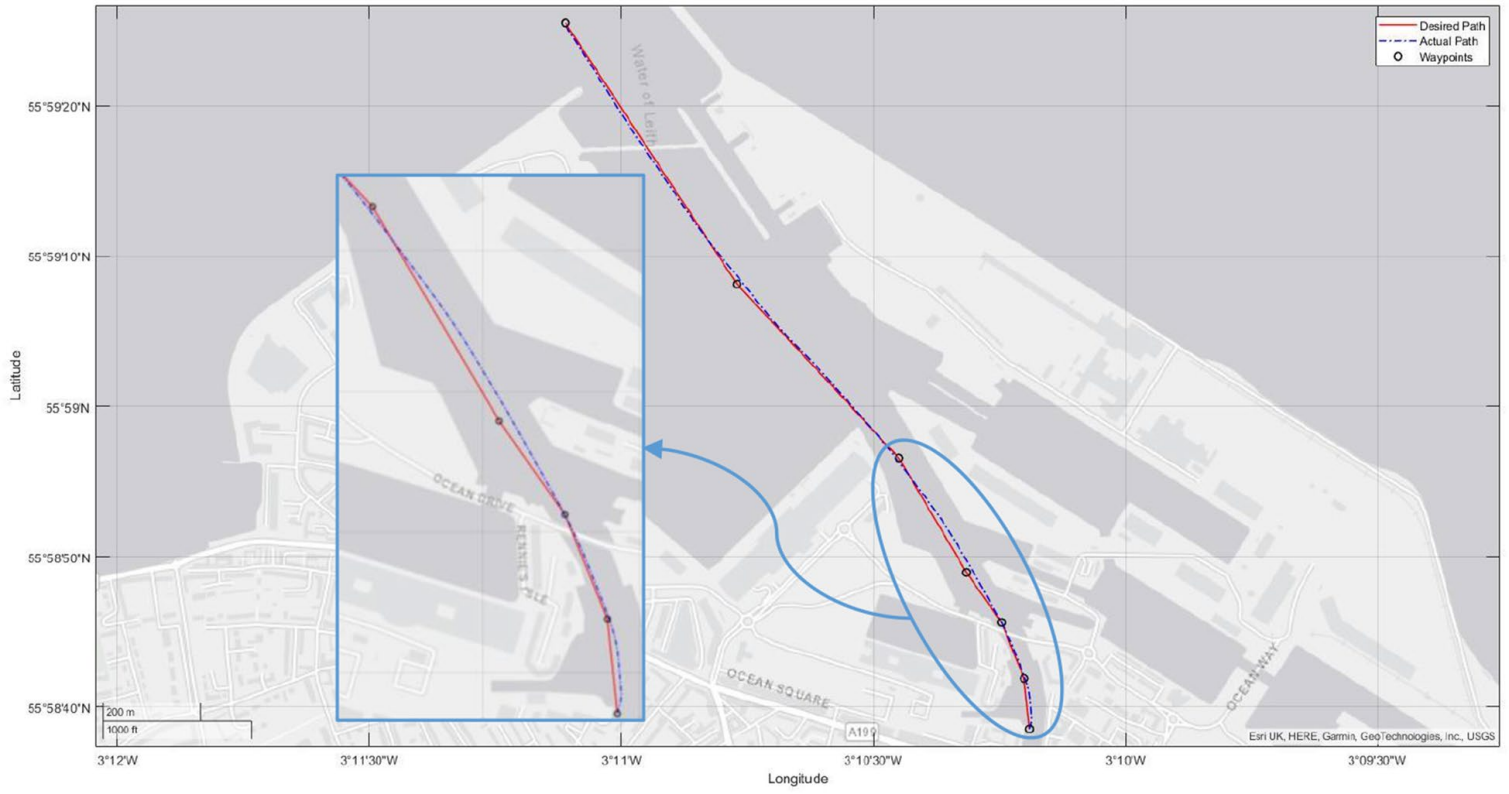
The trajectory of the ship.

**Figure 6:**
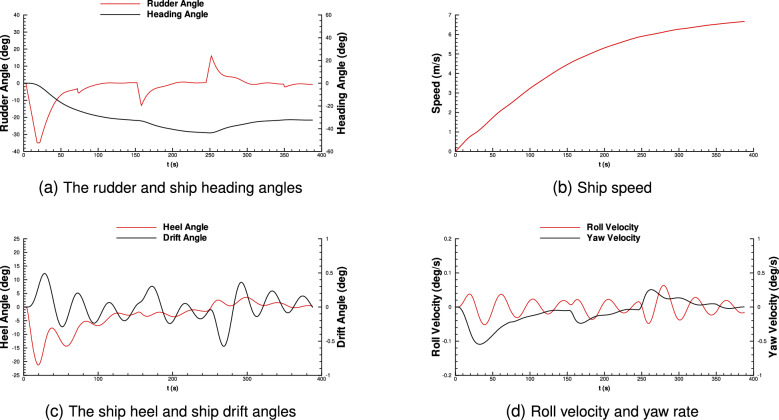
Time-based simulation results for ship maneuvering performance.

As shown in Fig. 5, the ship can take hard turns along the narrow channel in the Port of Leith with minimum overshoot due to her ability to control her actuators effectively. The heel angle does not exceed 22 degrees during the courses, while the roll velocity is between ${\mp }$0.1 degrees. Additionally, the maximum drift angle is lower than 0.6 degrees, and yaw velocity remains between ${\mp }$0.5 degrees per second. The shipping speed is steadily stable around 6-7 m$\setminus$s. The rate stability rises along the wide channel and becomes stable during the narrow channel. Moreover, the rudder and heading angles of the ship can be seen in Fig. 6.

Results show that the 1760.65 m actual distance was covered in 388 seconds; it significantly improved over the 410.52 seconds needed for the traditional approach. Our proposed model prevents excessive motions of the ship by minimizing the usage rates of its actuators. The ship stays on the path with minimum overshoot, and the distance covered by the ship is only 6 m longer than the actual path, while it is 110 m with the traditional approach. Implementing our solution saves up to 135 USD per hour, 3.24 thousand USD per day, and 1.18 million USD per year, while also reducing financial expenditure, protecting the environment by minimizing fuel consumption, and reducing greenhouse gas emissions, resulting in significant environmental benefits. All these results reveal the effectiveness of the proposed model implemented on the seaport and ship.

## Discussion

While the proposed TwinPort architecture offers a comprehensive port management system for smart seaports, several open issues must be addressed. For instance, drone-assisted data collection may pose challenges related to privacy concerns and the need to comply with regulatory frameworks. Additionally, implementing DT and drone-assisted data collection technologies may require a significant investment in infrastructure and specialized training for port staff and ship crews. Moreover, using wireless charging stations for drones may require considerable financing, and their reliability needs to be validated. Additionally, drones’ range and battery life may need to be improved regarding their ability to cover the required areas and collect data effectively.

Given the emphasis on precise ship maneuvering in our research, our data collection process exclusively considers the collection of essential ship details, including measurements such as length, breadth, draft, and other pertinent information. We designed a basic data collection protocol to this end; however, the environmental, distance, motion, chemical, and similar sensors can be added to the drone system to get overall data collection for smart seaports.

In this study, we assumed no conflict in data sharing for precise ship maneuvering between ship companies and port authorities. However, regional regulations and company interests may prevent this solution from being integrated. Some special legal arrangements may be required to solve such problems. Furthermore, regulatory and legal issues related to data sharing between ship companies and port authorities may challenge the implementation of our proposed architecture. Special arrangements must be made to ensure that data sharing is seamless and secure while complying with regional regulations and company interests. Therefore, further research is required to address these open issues and ensure the practical implementation of our proposed architecture in real-world scenarios.

## Conclusions

In conclusion, accurate ship navigation is critical in congested seaport areas to prevent delays, downtime, and collisions that can cause significant damage to property and loss of life. To address this issue, our paper proposed the TwinPort architecture, which combines digital twin technology and drone-assisted data collection to provide precise ship maneuvering in the maritime sector. Our recommendation engine ensures accurate ship navigation within a smart port during docking. The experimental results indicate that our solution improves trajectory performance by approaching the desired shortest path, reduces financial expenditures significantly, and minimizes fuel consumption, thereby reducing carbon emissions and protecting the environment.

## Data Availability

The datasets used and analysed during this study are available from the corresponding author on reasonable request.
